# Preventing death following unsafe abortion: a case series from urban Uganda

**DOI:** 10.1016/j.xagr.2021.100039

**Published:** 2022-02

**Authors:** Imelda Namagembe, Annettee Nakimuli, Josephat Byamugisha, Ashley Moffett, Abigail Aiken, Catherine Aiken

**Affiliations:** 1 Department of Obstetrics and Gynaecology, Makerere University and Mulago National Referral Hospital, Kampala, Uganda (Drs Namagembe, Nakimuli, and Byamugisha); 2 Department of Pathology and Centre for Trophoblast Research, University of Cambridge, Cambridge, United Kingdom (Dr Moffett); 3 Lyndon B. Johnson School of Public Policy, The University of Texas at Austin, Austin, TX (Dr A Aiken); 4 Department of Obstetrics and Gynaecology, University of Cambridge, The Rosie Hospital and National Institute for Health Research Cambridge Biomedical Research Centre, Cambridge, United Kingdom (Dr C Aiken)

**Keywords:** maternal death, postabortion care, sepsis, unsafe abortion, uterine perforation

## Abstract

**BACKGROUND:**

Maternal deaths from unsafe abortion continue to occur globally, with particularly high rates in Sub-Saharan Africa where most abortions are classified as unsafe. Maternal death reviews are an effective part of cohesive strategies to prevent future deaths while abortion remains illegal.

**OBJECTIVE:**

This study aimed to conduct maternal death reviews for all deaths occurring following unsafe abortion during the study period, to assess preventability, and to synthesize key learning points that may help to prevent future maternal deaths following unsafe abortions.

**STUDY DESIGN:**

Full case reviews of all maternal deaths (350 cases from Jan 2016 to Dec 2018) at the study center (a national referral hospital in urban Uganda) were conducted by specially trained multidisciplinary panels of obstetricians and midwives. We extracted the reviews of women who died following unsafe abortions (13 [2.6%]) for further analysis.

**RESULTS:**

Most maternal deaths owing to unsafe abortion were found to be preventable. The key recommendations that emerged from the reviews were (1) that clinicians should maintain a high index of suspicion for delayed presentation and rapid decompensation in cases where unsafe abortion has occurred, (2) that a low threshold for early intravenous antibiotic therapy should be applied, and (3) that any admission with complications following an unsafe abortion merits review by an experienced clinician as soon as possible.

**CONCLUSION:**

Postabortion care is part of essential emergency medical care and should be provided with high standards, especially in areas where there is limited or no legal access to abortion care. Implementing the recommended learning points is likely to be feasible even in low-resource obstetrical settings and, given the high rates of preventability found in maternal deaths owing to unsafe abortion, is likely to be effective.

**AJOG Global Reports at a Glance**

**Why was this study conducted?**

This study aimed to conduct detailed maternal death reviews for cases of unsafe abortion in a low-resource obstetrical setting and generate learning points.

**Key findings**

Clinicians should maintain a high index of suspicion for delayed presentation and rapid decompensation in women who have undergone an unsafe abortion. A low threshold for early intravenous antibiotic therapy and involvement of experienced clinicians as soon as possible are recommended.

**What does this add to what is known?**

Most deaths from unsafe abortion are potentially preventable with prompt and adequate management. The learning points generated are not resource intensive and are thus suitable for application in a low-resource obstetrical setting.

## Introduction

More than 120 women worldwide die every day following unsafe attempts to induce abortion.^1^ Most of these deaths occur in low-resource settings and jurisdictions where there are highly restrictive laws preventing access to safe and legal abortions.^1^ In East Africa, more than 75% of abortions are performed using methods classified by the World Health Organization as unsafe.^2^ Much global research in this area has focused on enabling people to access contraception and safe abortion care,^3^ including expanding access to manual vacuum aspiration procedures,^4^ making self-managed medication abortion more available via telemedicine,^5^ and establishing harm reduction and accompaniment models, such as hotlines, socorristas, and clinician-led support.^6^^,^^7^ However, despite these efforts, many women worldwide remain uninformed about or unable to access any safe method of ending a pregnancy. In East Africa, women who undergo unsafe abortions have a 7% risk of experiencing life-threatening complications^8^ and a 1 in 200 risk of dying because of the procedure.^1^ Therefore, it is imperative to understand how to maximize the chance of survival for women suffering medical complications from unsafe abortion. Postabortion care remains an essential emergency medical intervention that should be supported worldwide^9^; however, it is by no means universally available.^10^

Several studies have examined firsthand narratives of women who accessed unsafe abortion, providing important insights into their circumstances and experiences.^11^^,^^12^ Although further studies provide insight into abortion complications experienced by women in low-resource settings,^13^, ^14^, ^15^ there is a lack of data specifically examining the clinical case narratives of women who have died following unsafe abortions. These narratives provide important learning for healthcare providers, in particular examining whether there were medical opportunities to prevent their deaths. Maternal death reviews, particularly in the context of other quality improvement work, are likely to be effective in reducing the risk of death and improving care.^16^^,^^17^

Here, we aimed to examine the cases of women who died because of unsafe abortion in urban Uganda. The study setting was a low-resource obstetrical environment where access to safe abortion is highly restricted; however, Ugandan national guidelines support the provision of postabortion care at both primary and referral level healthcare facilities.^18^ We reported the synthesized learning points from the cases of all women who died following unsafe abortions in a tertiary obstetrics center throughout a 3-year period, drawing out important lessons for future care.

## Materials and Methods

We conducted a full case review of all maternal deaths at the study center between January 1, 2016, and December 31, 2018. During this period, 401 maternal deaths were identified in the register of maternity admissions, which was cross-checked against all mortuary records for women of reproductive age. Moreover, 51 cases were excluded as the medical notes were not identifiable or were incomplete or maternal death was not confirmed on the case review. Overall, 350 maternal deaths were included in the study cohort. Furthermore, we extracted the cases of women who died following unsafe abortions (13 [2.6%]) for further analyses.

The Mulago-Kawempe National Referral Site is the tertiary referral center for obstetrics in Uganda with between 25,000 and 32,000 deliveries per year.^19^ The center is one of the busiest maternity units in Africa and offers comprehensive obstetrics and gynecology services round the clock. The average number of maternal deaths at the center per year is approximately 136 to 140.^20^ The hospital is located in Kampala and serves a local low-resource urban population in addition to receiving referrals from other parts of Uganda.

All case notes were identified from the institutional records and retrieved by the study team. Each medical record was reviewed by members of a local multidisciplinary review team convened for the specific purpose of this study. All clinicians who participated in review panels (12 obstetricians and 8 highly experienced midwives) received specific training in maternal death case review by the study team and all signed a confidentiality agreement before reviewing any cases. All review panel members had received previous training in maternal death surveillance and response by the Ugandan Ministry of Health within 2 years and had some previous experience of conducting such reviews. Study-specific training was conducted at face-to-face courses, each lasting 1.5 working days. Each course was led by members of the research team and involved multidisciplinary groups of 5 participants (usually 3 obstetricians and 2 midwives). A training slide was developed for didactic teaching, followed by group discussions and a practice review of deidentified case records to ensure consistent standards and familiarity with the research data collection tool. The research tool used for data collection was adapted from the standardized Ugandan national maternal death audit or review form with extra questions added to assess the preventability of death and detail any delays in care. The training involved specific practice on consensus building within multidisciplinary groups on these aspects of the death reviews.

All identifying information of both the patient and medical team were obscured from the review panel. Every panel involved at least 1 obstetrician and 1 experienced midwife who were familiar with usual clinical practice and facilities at the study center. The study team ensured that no clinician reviewed any case in which they had any personal involvement.

The review panels categorized each case according to key medical and demographic characteristics and produced a detailed narrative summary for each patient. Furthermore, the review panel recorded their opinion on the preventability of each death and any missed opportunities in the care provided. In the absence of postmortem findings, all available sources of evidence were examined by the review panels to determine an accurate representation of the causes contributing to the death as specified by the International Classification of Diseases (ICD). The main source of information was the medical notes, a particularly detailed examination of the medical history, clinical findings, and contemporaneously recorded diagnoses of the treating clinicians. These were interpreted by the review panels given their extensive context-specific clinical experience. Moreover, the panel noted the diagnoses given on the maternal death audit forms (for those deaths audited). In several cases, the panel could consider documented collateral history (usually from a relative or friend) for patients who were admitted in critical condition plus some aspects of verbal autopsy from relatives and friends. Additional opinions on these aspects were sought where appropriate from other expert local clinicians—for example, obstetrical anesthetists or physicians. Each case summary was further independently reviewed by an international obstetrician who visited the study center on several occasions to become familiar with local contextual factors.

We presented summary statistics for the case reviews where death was because of unsafe abortion. Furthermore, cases were presented as anonymized illustrative narratives. The key elements of each case have been preserved, but some details have been changed to preserve the anonymity of the patient and small number suppression has been applied to demographic data to ensure anonymity where relevant.

The study was approved by the Makerere University Higher Degrees School of Medicine Research and Ethics Committee (#REC Ref 2018-001) and the Uganda National Council of Science and Technology (reference number SS4797).

## Results

### Demographics

The age range of the 13 women who died because of unsafe abortion was between 17 and 35 years, with a median age of 26 years (Table). Of the 13 women, 6 were married (although several of these were brought to the hospital by a family member other than their husband), and the rest were unmarried or of unknown relationship status. Moreover, 11 women had previously delivered children. The 13 maternal deaths examined in the study left a total of 28 children of various ages motherless.

**Table:** Characteristics of the mothers who died from complications of unsafe abortion (2016–2018)

Maternal characteristic	Frequency (n=13)	Proportion (%)	
Age (y)	17–24	5	38.5
	25–29	4	30.8
	≥30	4	30.8
Marital status	Married	6	46.2
	Unmarried or unknown	7	53.8
Distance travelled (km)	≤20	9	69.2
	˃20	4	30.8
Referral status	Referred	7	53.8
	Not referred	6	46.2
Admission to time of death (h)	<24	5	38.5
	>24	8	61.5
Delays in care	Yes	9	69.2
	No	4	30.8
Clinical condition on admission	Not critically ill	5	32.5
	Critically ill	8	61.5

Key aspects of care relating to deaths from unsafe abortion were identified by the review panel. We presented each below, using both context from all 13 cases and illustrative narratives.

### Accessing medical care

Eight women lived within 10 km from the study center; however, the maximum distance traveled to reach the study center was 125 km. Seven women initially presented to another healthcare facility following their abortion and were subsequently transferred to the study center for escalated care.

In most cases (9/13), there was a delayed presentation of medical care, with women spending between 2 days and 2 weeks at home with various symptoms, including severe vaginal bleeding, purulent vaginal discharge, and abdominal pain before seeking help.

On arrival at Mulago, 8 of the women were assessed by the admitting clinical team to be already critically ill or in extremis. The remainder were classed as either somewhat or significantly compromised. Of the 13 women, 8 had systolic blood pressures recorded on arrival at the hospital, which ranged from unrecorded to 102. All of the women who died had a respiratory rate of >20 cpm on admission.

The time between admission and death varied between 10 minutes and 6 days, with a median admission duration of 25 hours. Of note, 5 women were dead within 24 hours of reaching the study center. The women died on both weekdays and weekends in the same relative proportions.

### Narrative 1: delayed presentation for care

“Mary was a 34-year-old unmarried woman who lived in the suburb of urban Kampala with her 4 children. She had experienced several previous pregnancy losses. When Mary found herself pregnant for the eighth time, she visited a woman known locally to provide illegal abortions. The day after the procedure, she had severe pain and vaginal bleeding; however, she did her best to manage the symptoms at home.

Four days later, Mary had become increasingly sick with ongoing vaginal bleeding and spiking high fevers. She attended a local clinic where septic shock was quickly diagnosed and urgent transfer to the study center was arranged. On arrival, she was in critical condition, with high fever, blood pressure of 82/67 mm Hg, and a respiratory rate of 40 cpm. She was extremely pale and peripherally cool. She was peritonitic on abdominal examination, and uterine perforation was suspected. Plans were made by the admitting team for surgical management, but Mary died from overwhelming sepsis less than an hour after admission, before receiving any treatment.”

### Diagnoses

The primary diagnosis in 9 of 13 women who died following unsafe abortion was sepsis, which was also a secondary diagnosis in other cases. Uterine perforation was diagnosed in 5 cases, either clinically or at laparotomy. In 4 women, the primary diagnosis was hemorrhage, which was a secondary diagnosis in 5 cases.

A small proportion of the cohort tested positive for HIV, including at least 1 case in whom this was diagnosed for the first time. The observed likelihood of HIV infection was likely to reflect the overall prevalence of HIV infection,^21^ although most women who died were not tested during their admission.

### Narrative 2: complex presentation following abortion

“Sarah was a 19-year-old unmarried mother of 1 child living in downtown Kampala. On finding herself pregnant, Sarah sought an illegal abortion. After the procedure, she became gradually unwell throughout 5 days, initially with vaginal bleeding and pain. Moreover, she developed fever, acute-onset breathlessness, and chest pain. She attended the study center complaining of severe difficulty in breathing.

At the study center, she was admitted by a resident who prescribed antibiotics for presumed sepsis and transfused a unit of blood for presumed hemorrhage. Sarah's vital signs were not recorded during her initial admission or thereafter; however, within 4 hours of arrival, she was unconscious and was producing blood-tinged sputum from her mouth and nose. She was not assessed by an attending-level doctor at this point or subsequently. Sarah died on day 5 of her admission from sepsis complicated by pulmonary embolism.”

### Treatments

Six women were managed with intravenous (IV) antibiotics, but several other women for whom antibiotics were planned died before they were given. Four women had surgical intervention, all of whom had exploratory laparotomies, with or without uterine evacuation. Fewer than 3 women received misoprostol for medical management of retained products of conception. Of note, 3 women were given oxygen therapy before death, and <3 women received IV fluids. Three women received blood transfusions.

### Narrative 3: surgical management

“Joyce was a 29-year-old woman who lived in a rural area approximately 20 miles outside Kampala with her husband and 2 children. When she found out that she was pregnant for the third time, she traveled to Kampala for a surgical abortion performed at an unlicensed clinic. After the procedure, Joyce became increasingly unwell with severe abdominal pain and distention. She tried to return to the clinic where the abortion was performed for help, but she was told to go to the study center instead.

On arrival at the study center, Joyce was assessed and found to have frank peritonitis. A plan was made for an exploratory laparotomy. There was a delay in performing the procedure because of the lack of operating facilities and a delay in commencing IV antibiotics. On opening her abdomen, the surgeon found that there was extensive blood and feces within the abdomen. Further exploration revealed a through-and-through perforation of the transverse colon. Despite surgical repair and antibiotic therapy, Joyce's condition deteriorated, and she died in the intensive care unit (ICU) 48 hours after admission from septic shock.”

### Missed opportunities in medical care

The review panels considered that all but 1 of the 13 abortion-related deaths might have been prevented in the postabortion care phase. In 7 cases, the panel considered that there was a good chance that death would have been prevented had no delay in medical care occurred and had optimal treatments been available. Delays before presentation at the study center contributed to 9 deaths, and delays in receiving appropriate treatments after reaching the study center contributed to 6 deaths. Four women died while either awaiting ICU beds, which were all occupied, or awaiting a surgical procedure.

Only 3 women received blood transfusions, despite hemorrhage being implicated as a factor in 8 deaths. In at least 1 case, the lack of blood was determined by the review panel to have been a critical factor that could have prevented death. Three women did not have their vital signs monitored appropriately. Furthermore, failures to achieve IV access and administer therapeutic oxygen were noted. Review by an experienced clinician was highlighted by the review panel as a factor that might have helped to prevent death in several cases.

### Narrative 4: lack of blood and intensive care unit support

“Patience was a 26-year-old woman who lived in a village close to Kampala with her husband, 5 children, and her parents. Patience attended an unlicensed local clinic where a nonsterile instrument was inserted into the uterus to induce abortion. After the procedure, Patience began to bleed heavily. She attempted to manage the bleeding at home, but eventually, when she fainted while standing, her mother insisted that she attend the study center for medical care.

By the time she arrived at the study center, Patience was in hypovolemic shock. IV access was difficult to obtain, and thus, she did not receive adequate initial fluid resuscitation. A uterine evacuation was performed with further bleeding and subsequent laparotomy in the operating theater, but there was no blood available to transfuse and no ICU bed available. Patience died from severe hemorrhage postoperatively.”

### Case investigations and review

Of the 13 deaths, 5 (38%) were reported to the Ugandan Ministry of Health within a year of death occurring. None were reported within the recommended 7-day period, and the median reporting time was 287 days. Four cases (31%) were the subject of a local audit or review, with a median time to review of 213 days. None of the cases had a postmortem performed.

## Discussion

Abortion remains illegal in Uganda outside of a limited set of circumstances when it may be performed by a physician to save maternal life or to preserve physical and mental health. Abortion access in Uganda was expanded in 2006 in the limited circumstances of rape, incest, and preexisting health conditions, such as HIV. However, in practice, abortion remains unobtainable for most Ugandan women^21^, ^22^, ^23^

Approximately 46,000 women worldwide die per year following unsafe abortions^24^; however, there are few published recommendations to increase postabortion survival. In our case series, all but one of the deaths following unsafe abortion were considered preventable in the postabortion care phase by the review panel of local clinicians, underlining the importance of disseminating learning points from these cases.

### Principal findings and results

As with many other causes of maternal death in low-resource settings, delays in medical care emerged as the first key theme in this review.^25^^,^^26^ The general barriers to women accessing reproductive healthcare in low-resource settings are well-documented elsewhere, including financial concerns, lack of transport, and caring responsibilities.^27^, ^28^, ^29^ However, tackling delays in the context of unsafe abortion poses additional specific challenges.

The Ugandan health policy supports the provision of postabortion care^30^; however, previous studies have shown conflicting views among healthcare professionals about how this should be provided^31^ and inconsistent availability of care.^31^^,^^32^ Women who have undergone unsafe abortions may fear judgment from healthcare workers^31^^,^^33^ and legal punishment, including prosecution.^34^ Furthermore, previous studies have suggested that abortion is highly stigmatized in Uganda.^35^, ^36^, ^37^

Delay in seeking medical care is likely to be exacerbated by the nature of the injuries sustained by women undergoing unsafe abortions. Most deaths in our case series were because of sepsis, which was compounded by uterine perforation in more than half of women. Sepsis and uterine perforation are often slowly developing clinical presentations, which young and otherwise fit women may tolerate well until late in the disease course when they decompensate rapidly.^38^ Plausibly, several women were included in this review who did not present for medical care until a week or more following an unsafe abortion did not themselves realize how unwell they were.

The second key emerging theme from our case series was delay in providing appropriate care after arrival at the appropriate level medical facility. Although several women were already unconscious and in critical condition on arrival, including 3 women who died within an hour of reaching the study center, we identified cases in which prompt intervention could reasonably have been expected to alter the outcome. The resource deficits detailed in our work were in keeping with previous work examining the capacity for postabortion care in Uganda, particularly accessing blood transfusion and maintaining surgical capacity.^18^ Limited ICU and operating theater capacity, alongside difficulty in accessing blood for transfusion, were common themes in maternal death reviews from other causes in low-resource settings.^20^^,^^39^^,^^40^ However, we additionally identified lack of experienced clinician review as contributing to the deaths from unsafe abortion. It is possible that this oversight is linked to the underrecognition of the high mortality associated with complications after unsafe abortions or may reflect an element of low prioritization of such cases by clinical staff.^31^, ^32^, ^33^

In almost all the cases we reviewed, sepsis was an important element and contributed to death even when it was not the primary cause. Therefore, it is reasonable to have a low threshold for early antibiotic therapy for any woman who presents unwell following unsafe abortion.

### Clinical implications

Healthcare providers must recognize that women who have undergone an unsafe abortion are likely to present late for medical care and may be much sicker than they initially seem.

All cases where unsafe abortion has occurred or is suspected should have consideration of IV antibiotic therapy, because of the high incidence of sepsis, organ perforation, and peritonitis associated with unsafe abortion.

Experienced clinical review should be sought urgently, as soon as possible after admission, given that women who have undergone unsafe abortion often present late and are already extremely unwell.

### Research implications

Performing similar studies in other low-resource obstetrical settings where unsafe abortion is common would enable cross-setting comparison and further refinement of our clinical recommendations for other contexts. Further review of unsafe abortion deaths following the implementation of our recommendations in the study center is a key future research goal.

### Strengths and limitations

Our study has several important advantages. These included the systematic approach to identifying and screening cases, which resulted in 87% of maternal deaths throughout a 3-year period being available for review. This has enabled the collation of a relatively large single-cause case series of maternal deaths. These are particularly valuable data in the context of the cause of death that is difficult to study and a healthcare context where data collection poses considerable logistical challenges. Moreover, it provides important insight into the process and mechanism of maternal death in a context where postabortion care is complicated to access, for example, <10% of primary healthcare facilities in Uganda can provide basic postabortion care.^18^

Limitations of our study included that data collection was confined to a single center. However, as the national referral center, the study center receives complex cases from all areas of Uganda. Fewer than 25% of referral facilities in Uganda reported having the capacity to provide comprehensive postabortion care.^18^ In the case series presented here, women were transferred to the study center from >100 km away. A further limitation was that the review panels were primarily composed of clinicians working in the same center. Although all data were fully anonymized and any clinician involved with a specific case was excluded from the review panel, the chance that an assessor may have been previously familiar with a case could not be completely excluded. However, this risk was somewhat mitigated by a scale in a center that delivers >25,000 women annually and experiences on average 2 to 3 maternal deaths per week. Further mitigation came from the involvement of independent clinicians from other Ugandan centers and international reviews of all cases.

A further limitation related to the relatively low number of cases identified. Previous studies suggested that abortion accounts for 8% to 9% of maternal deaths,^24^ compared with 3% in our study. This may be the result of the high degree of stigma associated with accessing abortion in Uganda, resulting in women dying without presenting for care or concealing information about abortion. We identified several other unexplained deaths in early pregnancy within our wider study that could potentially have been attributable to unsafe abortion but had no definite information to classify them as such.

## Conclusions

Regardless of the legal status of abortion in any healthcare setting, there is an ethical imperative to provide safe postabortion care.^41^ There is a paucity of evidence from maternal death reviews internationally dealing specifically with deaths associated with unsafe abortion and lack of high-quality postabortion care. We presented recommendations based on detailed maternal death reviews of women who died following unsafe abortions in a low-resource setting. Our key recommendations for managing women who present following unsafe abortion were that (1) clinicians should maintain a high index of suspicion that unsafe abortion may have occurred and be alert to the possibility of delayed presentation and rapid decompensation in these cases, (2) a low threshold for early IV antibiotic therapy should be applied, and (3) admission with complications of an unsafe abortion merits review by an experienced clinician as soon as possible. Educating healthcare workers and community engagement about existing local laws regarding abortion may help to reduce stigma and encourage earlier access to postabortion care.
